# Development of a prediction rule for incomplete vaccination among children in Indonesia

**DOI:** 10.1186/s12889-025-23109-0

**Published:** 2025-05-24

**Authors:** Sofa D. Alfian, Rizky Abdulah, Eelko Hak

**Affiliations:** 1 Department of Pharmacology and Clinical Pharmacy, Faculty of Pharmacy, Universitas Padjadjaran, Jl. Raya Jatinangor, KM 21, Jatinangor, Sumedang Indonesia; 2 Drug Utilization and Pharmacoepidemiology Research Group, Centre of Excellence for Pharmaceutical Care Innovation, Universitas Padjadjaran, Jatinangor, Indonesia; 3 Center for Health Technology Assessment, Universitas Padjadjaran, Jatinangor, Indonesia; 4 Unit of PharmacoTherapy, -Epidemiology, & -Economics, Groningen Research Institute of Pharmacy, University of Groningen, Groningen, The Netherlands

**Keywords:** Prediction score, Children vaccination, Incomplete vaccination, Indonesia

## Abstract

**Background:**

Childhood vaccination is a fundamental public health intervention, playing an essential role in improving health outcomes and preventing serious infections. Despite proven benefits of vaccination programs, its coverage in Indonesia remains inadequate over the years. Therefore, this study aims to develop a prediction rule using intrapersonal, interpersonal, organizational, community, and policy-related factors to distinguish between Indonesian children < 2 years at high and low risk of incomplete vaccination.

**Methods:**

The prediction rule was developed using cross-sectional data from the 2017 Indonesia Demographic Health Survey. Data on vaccination status was obtained from a vaccination card, which was filled out by health care providers during vaccination. Multivariable logistic regression was applied to develop a prognostic score based on the regression coefficients of associated parental intrapersonal, interpersonal, organizational, community, and policy-related factors. Discrimination of the model was assessed with Receiver Operating Characteristic (ROC) curve.

**Results:**

The sample population in this study comprised 3,790 respondents, and 2,414 (63·7%) were incompletely vaccinated. Several factors such as a mother at young age, absence of a mobile telephone, limited antenatal care attendance, absence of postnatal checks within two months after birth, had not received tetanus vaccination during pregnancy, and low socio-economic status were independently associated with incomplete vaccination. The area under curve (AUC) of the model was 0·67, which showed moderate discrimination, but was acceptable. Using a cut-off score of > 20 points, only half of the parents with a high probability of incompletely vaccinated children are selected with a sensitivity of 60% and specificity of 64%, and only 41% of parents with incompletely vaccinated children are missed.

**Conclusion:**

This novel, easy-to-use prediction rule could be a useful tool to complement current strategies and further encourage tailored vaccine uptake interventions, particularly to parents with a high chance of incompletely vaccinated children in Indonesia.

**Supplementary Information:**

The online version contains supplementary material available at 10.1186/s12889-025-23109-0.

## Background

Childhood vaccination is an essential public health intervention, significantly enhancing health outcomes and preventing serious infections [1]. The acknowledged cost-effectiveness of the intervention in protecting individuals from various diseases and reducing associated morbidity and disability underscore its significance [2, 3]. Furthermore, a significant increase in life expectancy has been attributed to an increase in full vaccination coverage rates among children [4].

Despite its established benefits, Indonesia struggles with insufficient coverage of basic childhood vaccination [5, 6]. Between 2015 and 2018, Indonesia witnessed a large drop in vaccine confidence [7], partly triggered by the complexities of vaccine hesitancy [8]. The completion rate of basic vaccination among children aged 12–23 months was only 59% in 2013, and did not increase in later years [9]. Furthermore, vaccination coverage varies across various provinces in Indonesia [9] and consistently falls short of the national target [10]. As of 2021, 28 out of 34 provinces failed to achieve the stipulated national target of 94% coverage [11]. A substantial proportion (40%) of the 3,264 children surveyed exhibited incomplete vaccination, with higher rates observed in both urban (45%) and rural areas (55%) [12]. Another report showed that 8 of the 34 provinces reported rates exceeding the 50% threshold [12]. Such low vaccine coverage can lead to major epidemics for respiratory infections such as COVID-19 [13], respiratory syncytial virus infection [14], influenza [15], or pneumonia [16] where herd immunity is needed.

In line with these findings, identifying factors associated with low childhood vaccination rates is essential for tailoring effective interventions and policies to enhance its coverage in Indonesia. Despite the multifaceted factors, previous studies were however focused on individual-related aspects influencing childhood vaccination [12, 17, 18], limited in sample size or not representative of the Indonesian children [19], limited factors were included as determinants of incomplete vaccination [12, 20], or studies focused on one type of childhood vaccination [21].

The application of classical statistical analysis techniques in previous studies [12, 17, 18, 22], which observed the determinants of incomplete vaccination in Indonesian children, could potentially limit their implementation in practical settings. To help healthcare providers identify the risks associated with incomplete vaccination among children, we need a prediction rule, a simple risk assessment tool based on an accurate, rapid, simple, and objective multivariable model that predicts vaccination status using patient information. Such a tool can be noninvasive [23], cost-effective [24, 25], and may allow for the immediate development of targeted interventions [23]. However, such a tool is currently lacking in Indonesia. Therefore, this study aims to develop a prediction rule using intrapersonal, interpersonal, organizational, community, and policy-related factors to distinguish between Indonesian children under two years of age at high and low risk of incomplete vaccination.

## Methods

This study followed the Transparent Reporting of a multivariable prediction model for Individual Prognosis or Diagnosis (TRIPOD) Guideline [26] for prediction model development for its reporting (Table S1, Supplementary data).

### Study design

This prediction rule was developed using cross-sectional data from the 2017 Indonesia Demographic Health Survey (IDHS) [18]. The IDHS was part of the worldwide Demographic and Health Surveys, a comprehensive multi-topic survey focusing on maternal and child health. Furthermore, it was conducted across all 34 provinces in Indonesia by the Indonesian National Family Planning Coordinating Board in collaboration with the Ministry of Health and Statistics Indonesia [27]. The survey adhered to ethical standards, and data and procedures, including questionnaires, were reviewed and approved by ICF Institutional Review Board [28]. Verbal informed consent was then obtained from each respondent. The dataset was publicly available and could be acquired for free upon registration from the DHS website (https://dhsprogram.com/).

### Study population and data collection

Data were obtained from children aged 12−23 months within the 5 years preceding the survey using two-stage stratified sampling. The vaccination datasets were obtained from the Women’s Questionnaire, which contained detailed information on the vaccination history of the last two children. The survey also collected data from parents, particularly among ever-married women on socio-economic and demographic characteristics of households and members. This information was then combined with the detailed mother-level responses to the child vaccination questions, which were obtained from face-to-face structured interviews. The vaccination status was obtained with a card, which was verified by the enumerator. Mothers who have lost their children’ vaccination cards were excluded.

### Potential factors associated with low vaccination coverage

Potential factors associated with the low vaccination coverage were based on prior studies [12, 17]. Furthermore, the Social-Ecological Model was adopted as the framework to identify high-risk parents on the basis of administrative information [29, 30]. The Social-Ecological Model offers a comprehensive framework integrating intrapersonal, interpersonal, organizational, community, and policy-related factors, providing a multilevel framework to scrutinize determinants of health behaviors [29, 30]. For example, children of women who attended less than four antenatal care sessions and resided outside the Nusa Tenggara region, Indonesia, were more likely to be incompletely vaccinated, irrespective of urban or rural residence [12]. Meanwhile, a lack of health insurance had a positive association in urban areas. Receiving a tetanus injection during pregnancy had a negative association in rural areas [12]. Other factors, such as the mother’s education [31], engagement with healthcare services [31], media exposure [20], and household wealth [18] were linked to complete childhood vaccination in Indonesia [31].

Intrapersonal factors in this study comprised the order of child birth in the family as determined by parity (1st, 2nd, 3rd, 4th or 5th, or ≥ 6th -born), location of delivery (home or facility delivery), mother’s age at birth, calculated by subtracting the mother’s interview age from the child’s age (15–24, 25–34, or 35–49), education level (no education, primary, secondary, or higher), literacy level (cannot read at all, able to read only part of a sentence, able to read the whole sentence, or blind/visually impaired), media exposure (how often mothers spend time on reading newspaper, listening to the radio, and watching television which was categorized into less than once a week and at least once a week), ownership of a mobile telephone (yes or no), occupation (not working, clerk service and sales person, agriculture and industrial worker, or professional and manager), total antenatal care sessions (0–3 or ≥ 4), postnatal examination within 2 months of childbirth (yes or no), and tetanus vaccination (yes or no).

The interpersonal factors examined in this study included partner’s level of education (no education, primary, secondary, or higher), occupation (not working, clerk service and salesperson, agriculture and industrial worker, or professional and manager), number of children aged < 5 years in the household (≤ 1, 2, or ≥ 3), and household wealth status (low, middle, or high). Furthermore, the household wealth status was calculated based on the ownership of selected assets, where these properties were assigned a factor score from a principal components analysis for further standardization with a mean of zero and a standard deviation of one. The population quintiles of these standardized scores were then categorized into three classes (lowest (40%), middle (20%), or highest (40%)) [12]. The procedures also comprised information concerning the individual responsible for making financial decisions within the household, including choices related to the husband’s earnings, decisions about the mother’s healthcare, and determinations regarding significant household expenditure. The available options were respondent alone, respondent and husband/partner, or husband/partner alone. Data on whether mothers obtained permission for medical assistance, secured the necessary funds for treatment, or expressed reluctance to seek medical help alone were also obtained. Furthermore, the available response options categorized these issues as either a big problem or not a big problem.

Organizational factors included the perception of distance to a health facility (big problem or not a big problem), while community-level factors included the region (Papua, Maluku, Sumatra, Java and Bali, Kalimantan, Sulawesi, or Nusa Tenggara) and the location of residence as rural or urban. The classification of rural or urban areas was based on the size of the population, percentage of agricultural workers, and accessibility to public facilities. The policy-level factors in this study were the ownership of a national health insurance card (yes or no).

### Outcome

The dependent variable of this study is incomplete basic childhood vaccination, which is defined as a child aged 12–23 months who had not received the recommended ten vaccination doses stipulated by the Indonesian government [32] (Table 1).

**Table 1:** The recommended ten vaccination doses for children as stipulated by the Indonesian government

Type of vaccine	Time of delivery
One dose of the Bacille Calmette-Guérin (BCG) vaccine for tuberculosis	at birth
One dose of hepatitis B vaccine	after delivery
One dose of polio vaccine	after delivery
Three doses of polio	8, 12, and 16 weeks of age
Three doses of pentavalent (diphtheria-tetanus-pertussis-hepatitis B and Haemophilus influenza type B) vaccines	8, 12, and 16 weeks of age
One dose of the measles vaccine	9 months

### Development of the model

**Fig. 1:**
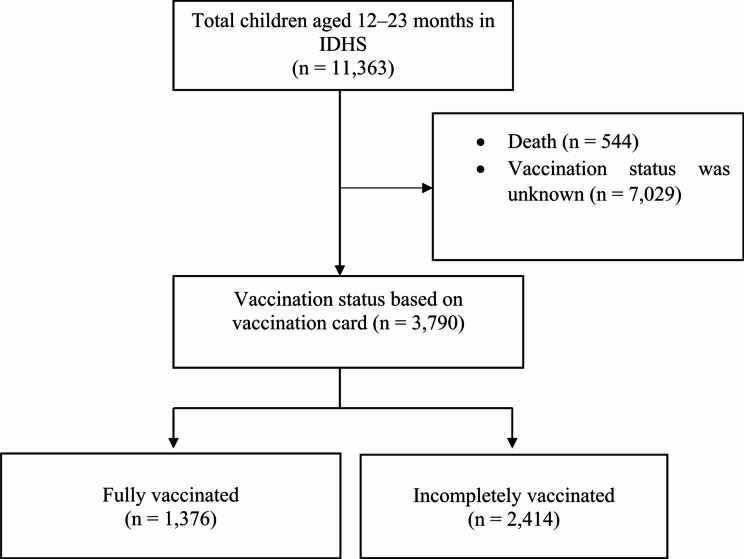
Flow diagram

To develop the model, data on a total of 3,790 children were used, as shown in Fig. 1. Descriptive statistics were presented as proportions for categorical variables to compare baseline characteristics between those with incomplete and complete vaccination coverage. Furthermore, the development of the prognostic model commenced with a univariate analysis, where the prognostic impact of each characteristic was assessed individually. The results were then expressed as odds ratios (ORs) accompanied by their corresponding 95% confidence intervals (CIs), utilizing logistic regression analysis. Since there was minimal missing data, a complete-case analysis was performed. In the next phase, multivariable logistic regression analysis was used to assess the prognostic impact of each characteristic simultaneously. Backward elimination procedure was performed to select variables that were related to the outcome, with *p <* 0.15 as a criterion for selection in univariate analysis [33, 34]. The results were reported as adjusted odds ratio (AOR). For each individual, the probability of the outcome from the final model (predicted probability) was calculated. The predicted and observed probabilities on the basis of the prediction rule were compared to obtain a sensitive and specific cut-off for the score.

### Evaluation of the model

The reliability of the multivariable logistic regression model was determined using the Hosmer-Lemeshow goodness-of-fit statistic [35]. The model’s discriminative ability was assessed with the area under the receiver-operating curve (ROC) [36]. The ROC was a plot of the true-positive rate (sensitivity) and the false-positive rate (1 − specificity) for each score. The area under the ROC could be explained as the probability that the logistic regression model assigned a higher probability of the outcome to a randomly chosen individual with an outcome compared to another person without an outcome. An area under the curve (AUC) estimates of 0.5 indicated the absence of discrimination, while a value of 1.0 showed perfect discrimination [36].

### Development and applicability of the prediction rule

To derive a simple-to-compute risk score and simplify interpretation, the regression coefficients of the derived multivariate model, which describe the size and direction of the relationship between a predictor and the response variable [37], were used to form the score value [38]. Furthermore, the predicted probability of outcome was determined as 1/(1 + e^− LP^), where the linear predictor (LP) = -0.4596 + (0.3618 x order of child birth is four) + (0.5679 x home delivery l) + (0.1798 x mother with age 14–24 years) + (0.3122 x mother not owns mobile phone) + (0.4893 x mother occupation as professional) + (0.8836 x less antenatal visit) + (0.1319 x did not have postnatal check) + (0.3702 x without tetanus injection during pregnancy) + (0.1036 x low wealth index status) + (0.7311 x province in Sumatera) + (0.5819 x province in Sulawesi) + (0.8738 x province in Maluku and Papua) + (0.1994 x individual responsible for making financial decisions within the household). The presence or absence of specific characteristics was coded as 1 (present) or 0 (absent). The regression coefficients were then multiplied by 20 and rounded to form the score value to simplify interpretation. Subsequently, the scores for individual prognostic variables were added to form the prognostic score (minimum 0 to maximum 119 points) for an outcome. For prognostic score cut-off points, the following test characteristics were calculated, such as positive predictive value, sensitivity, specificity, proportion of outcomes missed (1 − sensitivity), and proportion of respondents having the cut-off score or higher. All statistical analyses were performed using a standard software package (Stata, version. 12.0; StataCorp).

## Results

### Characteristics of respondents

Among the 11,363 children data surveyed, 3,790 met the inclusion criteria, where 2,414 (63.7%) were incompletely vaccinated, as shown in Fig. 1.

In terms of intrapersonal factors, the majority of mothers were aged 25−34 years (52.4%), graduated from secondary high school (76.0%), not working or were working as a clerk or in an agricultural sector (91.2%), and were in low to middle class of wealth status (83.4%) (Table S2, Supplementary data). Regarding interpersonal factors, the majority of them had partners with higher education (71.3%), as shown in Table S2, Supplementary data. Furthermore, in terms of organizational factors, the majority had no problem regarding the distance to the health facility (88.7%). For community-level factors, the majority were living in the provinces of Java and Bali (31.0%), and living in rural areas (50.6%). From a policy-level perspective, most of the respondents were being covered by health insurance (63.3%), as shown in Table S2, Supplementary data.

### Factors associated with incomplete vaccination

In univariate analysis, mother’s age, highest educational level, literacy, own a mobile phone, occupation, perception of distance to health facility, order of child birth, delivery location, number of antenatal care, postnatal checks, tetanus vaccination during pregnancy, total children, household wealth status, residency, province, individual responsible for making financial decisions within the household and whether mother secured the necessary funds for treatment were selected as potential factors associated with incomplete childhood vaccination, as shown in Table S3, Supplementary data.

In the multivariate analysis, several factors remained significantly independently associated with incomplete vaccination, for example, younger age of the mother (AOR = 1.26; 95%CI = 1.05–1.35; *p*-value = 0.014), attempting home delivery (AOR = 1.76; 95%CI = 1.41–2.20; *p*-value = 0.000), not owning a mobile phone (AOR = 1.30; 95%CI = 1.05–1.61; *p*-value = 0.015), working as professionals or manager (AOR = 1.74; 95%CI = 1.31–2.32; *p*-value = 0.000), only had three or less sessions of antenatal care (AOR = 2.44; 95%CI = 1.74–3.42; *p*-value = 0.000), did not receive tetanus injection during pregnancy (AOR = 1.43; 95%CI = 1.20–1.71; *p*-value = 0.000), had lower to middle wealth index (AOR = 1.23; 95%CI = 1.02–1.48; *p*-value = 0.034), and residency in the provinces of Sumatera (AOR = 2.00; 95%CI = 1.63–2.45; *p*-value = 0.000), Sulawesi (AOR = 1.70; 95%CI = 1.34–2.16; *p*-value = 0.000), and Maluku and Papua (AOR = 2.24; 95%CI = 1.59–3.16; *p*-value = 0.000) (Table 2).

**Table 2:** Results of multivariate analyses of incomplete vaccination status based on vaccination card (*N* = 3,790)

No	Characteristics	Fully Vaccinated (*n* = 1,376; 36.3%)	Incompletely Vaccinated (*n* = 2,414; 63.7%)		Multivariate^#^		
				Adjusted Odds Ratio (95% CI)			*p*-value
1	**Mother’s age (in years)**						
	14–24	365 (26.50)	649 (26.90)	1.26 (1.05–1.35)			0.014*
	25–34	748 (54.40)	1239 (51.30)	Ref			
	35–48	263 (19.10)	526 (21.80)	1.09 (0.88–1.35)			0.439
	Missing	0 (0)	0 (0)				
2	**Mother’s highest educational level**						
	No or primary education	279 (20.28)	630 (26.10)	1.08 (0.89–1.38)			0.378
	Secondary and higher education	1097 (79.72)	1784 (73.90)	Ref			
	Missing	0 (0)	0 (0)				
3	**Mother’s literacy**						
	Cannot read at all	39 (2.83)	155 (6.42)	1.22 (0.75–1.97)			0.418
	Able to read	1334 (96.95)	2252 (93.29)	Ref			
	Missing	3 (0.22)	7 (0.29)				
4	**Mother owns a mobile telephone**						
	No	235 (17.08)	625 (25.89)	1.30 (1.05–1.61)			0.015*
	Yes	1138 (82.70)	1788 (74.07)	Ref			
	Missing	3 (0.22)	1 (0.04)				
5	**Mother’s occupation**						
	Not working	760 (55.23)	1241 (51.41)	Ref			
	Clerk, service, and sales person	362 (26.31)	589 (24.4)	1.10 (0.91–1.32)			0.324
	Agriculture and industrial worker	149 (10.83)	356 (14.75)	1.16 (0.90–1.48)			0.254
	Professional and manager	105 (7.63)	228 (9.44)	1.74 (1.31–2.32)			0.000*
	Missing	0 (0)	0 (0)				
6	**Mother’s media exposure**			Not included			
	Less than once a week	657 (47.75)	1184 (49.05)				
	At least once a week	719 (52.25)	1230 (50.95)				
	Missing	0 (0)	0 (0)				
7	**Mother’s perception of distance to health facility**						
	Big problem	123 (8.94)	303 (12.55)	1.17 (0.89–1.55)			0.261
	Not a big problem	1253 (91.06)	2109 (87.37)	Ref			
	Missing	0 (0)	2 (0.08)				
8	**Order of child birth in the family**						
	1st, 2nd, and 3rd	1224 (88.95)	1958 (81.11)	Ref			
	4th	102 (7.41)	249 (10.31)	1.32 (1.00–1.79)			0.078*
	≥ 5th	50 (3.63)	207 (8.57)	1.56 (0.79–3.07)			0.199
	Missing	0 (0)	0 (0)				
9	**Delivery location**						
	Home delivery	189 (13.74)	708 (29.33)	1.76 (1.41–2.20)			0.000*
	Facility delivery	1187 (86.26)	1706 (70.67)	Ref			
	Missing	0 (0)	0 (0)				
10	**Number of antenatal care (in sessions)**						
	0−3	60 (4.36)	348 (14.42)	2.44 (1.74–3.42)			0.000*
	≥ 4	1282 (93.17)	1921 (79.58)	Ref			
	Missing	34 (2.47)	145 (6.01)				
11	**Postnatal checks within 2 months**						
	No	382 (27.76)	774 (32.06)	1.15 (0.97–1.36)			0.010*
	Yes	955 (69.40)	1468 (60.81)	Ref			
	Missing	39 (2.83)	172 (7.13)				
12	**Previous tetanus vaccination during pregnancy**						
	No	306 (22.24)	747 (30.94)	1.43 (1.20–1.71)			0.000*
	Yes	1016 (73.84)	1493 (61.85)	Ref			
	Missing	54 (3.92)	174 (7.21)				
13	**Partner’s education level**						
	No education or primary/secondary education	339 (24.64)	648 (26.84)	Ref			
	Secondary and higher education	1008 (73.26)	1694 (70.17)	1.07 (0.88–1.31)			0.505
	Missing	29 (2.11)	72 (2.98)				
14	**Partner’s occupation**			Not included			
	Clerk, agriculture, not working	1243 (90.33)	2186 (90.56)				
	Professional and manager	133 (9.67)	228 (9.44)				
	Missing	0 (0)	0 (0)				
15	**Total children under 5 years old**						
	1 and 2	974 (70.78)	1485 (61.52)	Ref			
	≥ 3	363 (26.38)	756 (31.32)	1.09 (0.89–1.35)			0.409
	Missing	39 (2.83)	173 (7.17)				
16	**Household wealth status**						
	Lower and middle	1079 (78.42)	2016 (83.51)	1.23 (1.02–1.48)			0.034*
	Rich	297 (21.58)	398 (16.49)	Ref			
	Missing	0 (0)	0 (0)				
17	**Covered by health insurance**			Not included			
	No	493 (35.83)	898 (37.20)				
	Yes	883 (64.17)	1516 (62.8)				
	Missing	0 (0)	0 (0)				
18	**Residency**						
	Urban	749 (54.43)	1122 (46.48)	Ref			0.837
	Rural	627 (45.57)	1292 (53.52)	1.02 (0.86–1.21)			
	Missing	0 (0)	0 (0)				
19	**Province**						
	Sumatera	259 (18.80)	716 (29.70)	2.00 (1.63–2.45)			0.000*
	Java and Bali	568 (41.30)	606 (25.10)	Ref			
	Nusa Tenggara	135 (9.80)	204 (8.50)	1.19 (0.90–1.59)			0.227
	Kalimantan	151 (11.00)	185 (7.70)	0.84 (0.64–1.11)			0.213
	Sulawesi	185 (13.40)	386 (16.00)	1.70 (1.34–2.16)			0.000*
	Maluku and Papua	78 (5.70)	317 (13.10)	2.24 (1.59–3.16)			0.000*
	Missing	0 (0)	0 (0)				`
20	**Individual responsible for making financial decisions within the household**						
	Respondent alone	649 (47.17)	1084 (44.90)	1.01 (0.86–1.19)			0.880
	Respondent and husband/partner	575 (41.79)	978 (40.51)	Ref			
	Husband/partner alone	120 (8.72)	267 (11.06)	1.24 (1.00–1.62)			0.117*
	Missing	32 (2.33)	85 (3.52)				
21	**Individual responsible for decisions about the mother’s healthcare**			Not included			
	Respondent alone	604 (43.9)	1032 (42.75)				
	Respondent and husband/partner	602 (43.75)	1050 (43.5)				
	Husband/partner alone	142 (10.32)	256 (10.6)				
	Missing	28 (2.03)	76 (3.15)				
22	**Determinations regarding significant household expenditure**			Not included			
	Respondent alone	202 (14.68)	360 (14.91)				
	Respondent and husband/partner	813 (59.08)	1448 (59.98)				
	Husband/partner alone	328 (23.84)	526 (21.79)				
	Missing	33 (2.40)	80 (3.31)				
23	**Whether mothers obtained permission for medical assistance**			Not included			
	Big problem	82 (5.96)	148 (6.13)				
	Not a big problem	1294 (94.04)	2265 (93.83)				
	Missing	0 (0)	1 (0.04)				
24	**Whether mother secured the necessary funds for treatment**						
	Big problem	210 (15.26)	416 (17.23)	Ref			
	Not a big problem	1166 (84.74)	1997 (82.73)	1.06 (0.84–1.34)		0.611	0.466
	Missing	0 (0)	1 (0.04)				
25	**Whether mother expressed reluctance to seek medical help alone**			Not included			
	Big problem	286 (20.78)	514 (21.29)				
	Not a big problem	1090 (79.22)	1899 (78.67)				
	Missing	0 (0)	1 (0.04)				

Note:

*: Statistically significant at *p*-value < 0.15

^#^: Hosmer-Lemeshow = 0.727; Pseudo-*R* = 0.0704

**Fig. 2:**
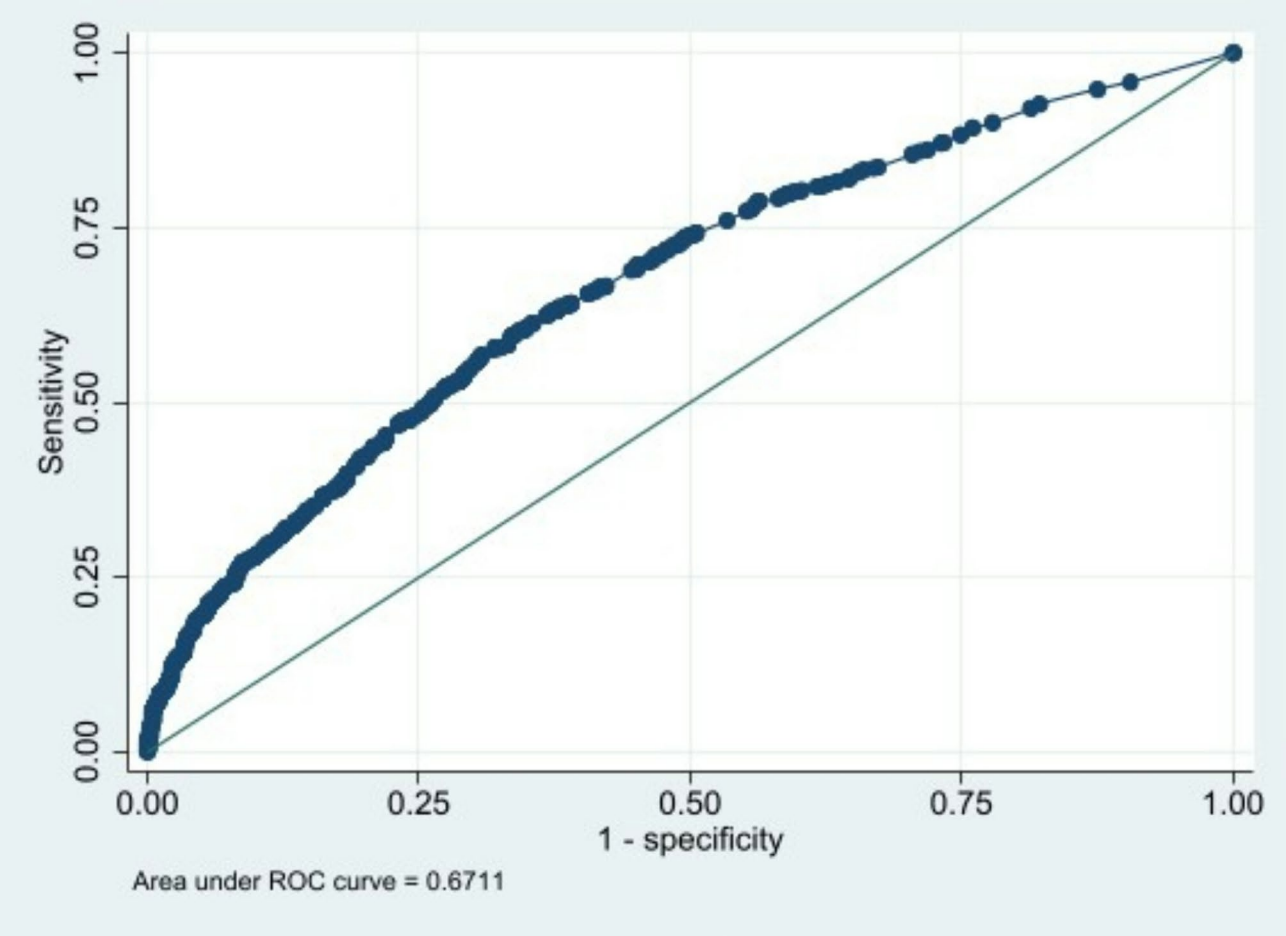
Receiver-operating curve (ROC) for the predicted probability

Furthermore, the performance of the final model was good (*p*-value = 0.727) based on the Hosmer-Lemeshow goodness-of-fit test. The AUC of the model was 0.67, which showed moderate discrimination, as shown in Fig. 2.

### Prediction rule

The prediction rule was derived from variables with *p*-value < 0.05 in the final multivariate model. Table 3 showed the result of the probability score of incomplete vaccination among respondents. Furthermore, mothers who were younger (15–24 years old) (score: 4), not owned mobile telephones (score: 6), worked as a professional and manager (score: 10), 4th child born in the family (score: 7), home delivery (score: 11), attending the maximum of three sessions of antenatal care (score: 18), not having postnatal check within two months (score: 3), having no previous tetanus vaccinations (score: 7), lower to middle economic level (score: 5), residency in Sumatera (score: 15), Sulawesi (score: 12), Maluku and Papua (score: 17), and letting the husband or partner to fully manage the household spending (score: 4) were factors that statistically significantly increased the probability for getting incomplete vaccination (Table 3).

**Table 3:** Prediction score for estimating the probability of incompletely vaccinated

No	Characteristics	Coefficient	Score ^a, b^
1	**Mother’s age (in years)**		
	14−24	0.1797	4
	25−34	0	0
	35–48	0	0
2	**Mother owns a mobile telephone**		
	No	0.3122	6
	Yes	0	0
3	**Mother’s occupation**		
	Not working	0	0
	Clerk, service, and sales person	0	0
	Agriculture and industrial worker	0	0
	Professional and manager	0.4893	10
4	**Order of child birth in the family**		
	1st, 2nd, and 3rd	0	0
	4th	0.3618	7
	5th or above	0	0
5	**Delivery location**		
	Home delivery	0.5679	11
	Facility delivery	0	0
6	**Number of antenatal care (in sessions)**		
	0−3	0.8835	18
	≥4	0	0
7	**Postnatal checks within 2 months**		
	No	0.1319	3
	Yes	0	0
8	**Previous tetanus vaccination**		
	No	0.3702	7
	Yes	0	0
9	**Household wealth status**		
	Low and middle	0.2502	5
	High	0	0
10	**Province**		
	Sumatera	0.7310	15
	Java, Bali	0	0
	Nusa Tenggara	0	0
	Kalimantan	0	0
	Sulawesi	0.5819	12
	Maluku and Papua	0.8738	17
11	**Individual responsible for making financial decisions within the household**		
	Respondent alone	0	0
	Respondent and husband/partner	0	0
	Husband/partner alone	0.1994	4

^a^ The prognostic score for a particular subject can be obtained by adding the scores for each applicable characteristic. The prognostic score correlates with the predicted probability which includes the regression coefficients of the derived multivariate model

^b^ ROC model: 0.6711

The prediction rule was derived from the final multivariate model, where a score was assigned to the absence or presence of each factor (Table 3). A prognostic score for each patient, interpreting the probability of getting incomplete vaccination, was calculated by adding scores of each relevant factor. For example, the prognostic score for a mother aged 22 years old who worked as a manager, received tetanus injection during pregnancy, attempted home delivery, and had two sessions of antenatal care was 43 (4 + 10 + 0 + 11 + 18), representing a higher probability of the child not being vaccinated that is almost two times higher than that of the lowest category (Table 4).

**Table 4:** Test characteristics of prediction score cutoff points

Prognostic Score Category	No (%) of Respondent	OP, (%)	RR	Cutoff Point	PPV, (%)	SE, (%)	SP, (%)	OM, (%)	Selection, (%)
0 to < 10	834 (22.0)	47.2	1.0	0	63.7	100.0	0	0	100.0
10 to < 20	1036 (27.3)	57.6	1.2	10	68.3	83.7	32.0	16.3	77.99
20 to < 30	1023 (26.9)	68.3	1.4	20	74.1	58.9	63.9	41.1	50.65
30 to < 40	502 (13.3)	76.3	1.6	30	80.7	30.0	87.4	70	23.66
40 to < 50	258 (6.8)	83.7	1.8	40	86.3	14.1	96.1	85.9	10.41
50 to < 60	80 (2.1)	88.8	1.9	50	91.2	5.2	99.1	94.8	3.6
60 to < 70	45 (1.2)	95.6	2.0	60	94.7	2.2	99.8	97.8	1.49
≥ 70	12 (0.3)	91.7	1.9	70	91.7	0.5	100.0	99.5	0.3

NOTE. OM, outcomes missed (proportion of outcomes occurring among respondents with a score less than the indicated cut-off point); OP, observed probability of outcome among respondents having a score within the indicated score category; PPV, positive predictive value for an outcome among respondents having a score higher or equal to the cut-off point; RR, relative risk (< 10 points is the reference); SE, sensitivity; Selection, the percentage of having a score higher or equal to the indicated cut-off point; SP, specificity

Test characteristics for each cut-off level of the prognostic score can be seen in Table 4. A cut-off score of 20 for defining higher risk had a sensitivity of 58.9% (59 of 100 outcomes occurred in this group, while 41 of 100 occurred among respondents with a score of < 20). With increasing cut-off levels for defining higher risk, the proportion of persons classified as lower risk would increase, but the proportion of outcomes occurring among the lower-risk persons would also increase. To optimize the trade-offs between sensitivity and specificity, we selected a score of ≥ 20 for defining higher risk (Table 4).

## Discussion

In this study, a prediction rule for quantifying the probability of incomplete vaccination among children was obtained, with moderate discriminating ability, and acceptability, using national-level data in Indonesia. Using a cut-off score of > 20 points, only half of the parents with a high probability of incompletely vaccinated children are selected with a sensitivity of 60% and specificity of 64%, and only 41% of parents with incompletely vaccinated children are missed.

Several factors incorporated in the prediction rule were also been established in previous studies as determinants for potential incomplete vaccination among children. For example, we observed that mothers aged 14 to 24 years were less likely to get their children vaccinated which is in line with previous studies [39, 40]. This could be explained by higher tendencies to doubt the effectiveness and safety of vaccination in this young age group, leading to low compliance levels [39, 40].

Mothers without a mobile phone had been associated with a higher probability of incomplete vaccination [41–43]. This could be explained by the lower media exposure, which may lead to a lower awareness of vaccination [44]. Thus, increasing mother’s initial adoption of media use, including mobile phones, may improve children’s vaccination status.

The fourth order of child born in the family appeared to be less likely to get fully vaccinated. This was in line with a study, where parents of children born later took less care of their health due to old age and the latter of birth order was reported to reduce the likelihood of on-time full vaccination compared to firstborns [45].

Children of mothers who attempted home delivery, had three or fewer sessions of antenatal care, and had no postnatal check within 2 months after birth, and had no previous tetanus vaccination during pregnancy had lesser probability to get their children fully vaccinated, which was similar to previous findings [46–56]. Facility delivery, antenatal care, and postnatal checks provided a better chance for mothers to communicate and get educated by healthcare workers [46, 47], which was associated with complete vaccination [46, 57, 58, 53–56]. 

Lower and middle household wealth status was reported to be associated with a low probability of the child not being vaccinated [59, 60]. While childhood vaccination services are provided without charge in Indonesia, indirect costs such as lost work days and transportation expenses may discourage parents from bringing their child to get vaccinated; thus, indirect costs may be more important to consider [61–63].

Residents of Sumatera, Sulawesi, Maluku, and Papua were more likely to be incompletely vaccinated, which is in agreement with the reported low coverage of vaccination (3.8–13.3%) in the provinces of North Sumatera, West Sumatera, West Kalimantan, Central Kalimantan, North Maluku, and Papua [10]. These disparities could be caused by a wide gap in the accessibility of healthcare facilities and human resources between the regions, as well as the limited operational budget allocated for the vaccination program at the local government level, which could potentially impact service delivery and coverage [64]. Furthermore, vaccine hesitancy and education about vaccines may differ significantly between the regions [65].

Households where only husbands or partners decided on the earnings and expenditures played a role in the probability of the children not receiving vaccines [59, 66]. Empowerment of women in decision-making was associated with a higher rate of childhood vaccination [59, 67–73]. The empowerment of women was further associated with their autonomy and capacity for making well-informed decisions, particularly when it comes to seek health information and preventive care for their children [74].

A higher level of occupation was associated with higher knowledge and awareness [52, 57, 58], leading to better compliance with vaccination [39]. However, in this study, mothers who worked as professionals and managers had a higher probability of not fully vaccinating their children. Other factors could facilitate compliance with vaccination, including religious reasons, personal beliefs or philosophical reasons, and safety concerns [75].

The developed prediction rule was expected to help healthcare providers to provide more cost-effective use of resources by selecting parents at high risk of incomplete child vaccination and incorporating the values of parents and children who need tailored interventions. Moreover, the rule was designed to serve as an assistive clinical tool to guide healthcare providers, and not to function as a directive tool that explicitly influenced their decision. This prediction rule could be ideally implemented by using a digital application in maternal clinics, community health centers, and hospitals. Furthermore, an overall risk score could be also calculated for each infant during the birth hospitalization and communicated to the outpatient care team. Outpatient practices and providers could use this information to tailor early visits and support families identified as high risk for incomplete vaccination as well as provide other current interventions. Evidence-based interventions are an option to meet the individual needs of each family. For example, strategies, such as transportation assistance [76], maternal education [77], and motivational interviewing [78] could be delivered to ensure equitable access to childhood vaccination services. The risk prediction tool also will alert healthcare providers to schedule additional or longer visits with high-risk families. This could prompt healthcare providers to address barriers to vaccination and initiate vaccine conversations.

To the best of our knowledge, this was the first study to develop a prediction rule for incomplete children vaccination in Indonesia. However, several limitations must be acknowledged, including the retrospective nature of this study, leading to susceptibility to potential information bias. A further prospective investigation is needed to externally validate this prediction rule, translate the results of the validated rule into practice, and assess its impact on healthcare providers’ behavior and clinical outcomes. Despite having obtained a large amount of information from parents, a prediction rule that was both highly sensitive (> 80%) and specific (> 80%) could not be developed. Nonetheless, the less-than-ideal performance of the rule is believed to be considerably better than the single judgment of a healthcare provider. In future research, the discrimination ability of the predictive rule may be further improved by incorporating more behavioral data, including attitudes and other parent-related data such as the age at which the child should get vaccinated, and the number of children, which were currently unmeasured factors in the administrative survey database.

## Conclusion

This study obtained a prediction rule for quantifying the probability of incomplete vaccination among children with moderate discriminating ability and acceptability. This novel, easy-to-use prediction rule could be a useful tool to complement current strategies and further encourage tailored vaccine interventions, such as early visits and additional or longer visits to address barriers to vaccination and initiate vaccine conversations, particularly with parents of young children with a high risk of incomplete vaccination in Indonesia.

## Electronic supplementary material

Below is the link to the electronic supplementary material.

Supplementary Material 1

## Data Availability

Data is provided within the manuscript or supplementary information files.
