# Supplementary material for: Development of a prediction rule for incomplete vaccination among children in Indonesia
Source: BMC Public Health. 2025 May 24;25:1915. doi: 10.1186/s12889-025-23109-0 (PMC12102914; doi:10.1186/s12889-025-23109-0)
Supplement: Supplementary file 1 — Supplementary Material 1 [file 12889_2025_23109_MOESM1_ESM.docx]

**SUPPLEMENTARY DATA**

**Table S1. TRIPOD Checklist: Prediction Model Development**

| **Section/Topic** | **Item** | **Checklist Item** | **Page** |
| --- | --- | --- | --- |
| **Title and abstract** | | | |
| Title | 1 | Identify the study as developing and/or validating a multivariable prediction model, the target population, and the outcome to be predicted. | 1 |
| Abstract | 2 | Provide a summary of objectives, study design, setting, participants, sample size, predictors, outcome, statistical analysis, results, and conclusions. | 2 |
| **Introduction** | | | |
| Background and objectives | 3a | Explain the medical context (including whether diagnostic or prognostic) and rationale for developing or validating the multivariable prediction model, including references to existing models. | 4 |
|  | 3b | Specify the objectives, including whether the study describes the development or validation of the model or both. | 5 |
| **Methods** | | | |
| Source of data | 4a | Describe the study design or source of data (e.g., randomized trial, cohort, or registry data), separately for the development and validation data sets, if applicable. | 6 |
|  | 4b | Specify the key study dates, including start of accrual; end of accrual; and, if applicable, end of follow-up. | 6 |
| Participants | 5a | Specify key elements of the study setting (e.g., primary care, secondary care, general population) including number and location of centres. | 6 |
|  | 5b | Describe eligibility criteria for participants. | 6 |
|  | 5c | Give details of treatments received, if relevant. | NA |
| Outcome | 6a | Clearly define the outcome that is predicted by the prediction model, including how and when assessed. | 9 |
|  | 6b | Report any actions to blind assessment of the outcome to be predicted. | NA |
| Predictors | 7a | Clearly define all predictors used in developing or validating the multivariable prediction model, including how and when they were measured. | 7 |
|  | 7b | Report any actions to blind assessment of predictors for the outcome and other predictors. | NA |
| Sample size | 8 | Explain how the study size was arrived at. | NA |
| Missing data | 9 | Describe how missing data were handled (e.g., complete-case analysis, single imputation, multiple imputation) with details of any imputation method. | NA |
| Statistical analysis methods | 10a | Describe how predictors were handled in the analyses. | 9-10 |
|  | 10b | Specify type of model, all model-building procedures (including any predictor selection), and method for internal validation. | 9-10 |
|  | 10d | Specify all measures used to assess model performance and, if relevant, to compare multiple models. | 9-10 |
| Risk groups | 11 | Provide details on how risk groups were created, if done. | NA |
| **Results** | | | |
| Participants | 13a | Describe the flow of participants through the study, including the number of participants with and without the outcome and, if applicable, a summary of the follow-up time. A diagram may be helpful. | 11 |
|  | 13b | Describe the characteristics of the participants (basic demographics, clinical features, available predictors), including the number of participants with missing data for predictors and outcome. | 11 |
| Model development | 14a | Specify the number of participants and outcome events in each analysis. | 11 |
|  | 14b | If done, report the unadjusted association between each candidate predictor and outcome. | 11 |
| Model specification | 15a | Present the full prediction model to allow predictions for individuals (i.e., all regression coefficients, and model intercept or baseline survival at a given time point). | 12 |
|  | 15b | Explain how to the use the prediction model. | 12 |
| Model performance | 16 | Report performance measures (with CIs) for the prediction model. | 12 |
| **Discussion** | | | |
| Limitations | 18 | Discuss any limitations of the study (such as nonrepresentative sample, few events per predictor, missing data). | 16 |
| Interpretation | 19b | Give an overall interpretation of the results, considering objectives, limitations, and results from similar studies, and other relevant evidence. | 14-16 |
| Implications | 20 | Discuss the potential clinical use of the model and implications for future research. |  |
| **Other information** | | | |
| Supplementary information | 21 | Provide information about the availability of supplementary resources, such as study protocol, Web calculator, and data sets. | 18 |
| Funding | 22 | Give the source of funding and the role of the funders for the present study. | 17 |

We recommend using the TRIPOD Checklist in conjunction with the TRIPOD Explanation and Elaboration document.

**Table S2. Characteristics of Respondents (N = 3,790)**

| **No** | **Characteristics** | **N (%)** |
| --- | --- | --- |
| **Intrapersonal factors** | | |
| 1 | **Mother's age (in years)** |  |
|  | 14−24 | 1014 (26.8) |
|  | 25−34 | 1987 (52.4) |
|  | 35–48 | 789 (20.8) |
|  | Missing | 0 (0) |
| 2 | **Mother’s highest educational level** |  |
|  | No or Primary Education | 909 (23·98) |
|  | Secondary And Higher Education | 2881 (76·02) |
|  | Missing | 0 (0) |
| 3 | **Mother’s literacy** |  |
|  | Cannot read at all | 194 (5·12) |
|  | Able to read | 3586 (94·62) |
|  | Missing | 10 (0·26) |
| 4 | **Mother owns a mobile telephone** |  |
|  | No | 860 (22·69) |
|  | Yes | 2926 (77·20) |
|  | Missing | 4 (0·11) |
| 5 | **Mother’s occupation** |  |
|  | Not Working | 2001 (52·8) |
|  | Clerk, Service, and Sales Person | 951 (25·09) |
|  | Agriculture and Industrial Worker | 505 (13·32) |
|  | Professional and Manager | 333 (8·79) |
|  | Missing | 0 (0) |
| 6 | **Mother’s media exposure** |  |
|  | Less than once a week | 1841 (48·58) |
|  | At least once a week | 1949 (51·42) |
|  | Missing | 0 (0) |
| 7 | **Order of child birth in the family** |  |
|  | 1^st^, 2^nd^, and 3^rd^ | 3182 (83·96) |
|  | 4^th^ | 351 (9·26) |
|  | 5^th^ or above | 257 (6·78) |
|  | Missing | 0 (0) |
| 8 | **Delivery location** |  |
|  | Home delivery | 897 (23·67) |
|  | Facility delivery | 2893 (76·33) |
|  | Missing | 0 (0) |
| 9 | **Number of antenatal care (in sessions)** |  |
|  | 0−3 | 408 (10·77) |
|  | ≥ 4 | 3203 (84·51) |
|  | Missing | 179 (4·72) |
| 10 | **Postnatal checks within 2 months** |  |
|  | No | 1156 (30·5) |
|  | Yes | 2423 (63·93) |
|  | Missing | 211 (5·57) |
| 11 | **Previous tetanus vaccination during pregnancy** |  |
|  | No | 1053 (27·78) |
|  | Yes | 2509 (66·2) |
|  | Missing | 228 (6·02) |
|  |  |  |
| **Interpersonal factors** | | |
| 1 | **Partner’s education level** |  |
|  | No Education/Primary/Secondary education | 987 (26·04) |
|  | Higher education | 2702 (71·29) |
|  | Missing | 101 (2·66) |
| 2 | **Partner’s occupation** |  |
|  | Clerk, Agriculture, or not working | 3429 (90·47) |
|  | Professional and manager | 361 (9·53) |
|  | Missing | 0 (0) |
| 3 | **Total children under 5 years old** |  |
|  | 1–2 | 2459 (64·88) |
|  | ≥ 3 | 1119 (29·53) |
|  | Missing | 212 (5·59) |
| 4 | **Household wealth status** |  |
|  | Low to Middle | 3095 (81·66) |
|  | High | 695 (18·34) |
|  | Missing | 0 (0) |
| 5 | **Individual responsible for making financial decisions within the household** |  |
|  | Respondent Alone | 1733 (45·73) |
|  | Respondent And Husband/Partner | 1553 (40·98) |
|  | Husband/Partner Alone | 387 (10·21) |
|  | Missing | 117 (3·09) |
| 6 | **Individual responsible for decisions about the mother's healthcare** |  |
|  | Respondent Alone | 1636 (43·17) |
|  | Respondent And Husband/Partner | 1652 (43·59) |
|  | Husband/Partner Alone | 398 (10·5) |
|  | Missing | 104 (2·74) |
| 7 | **Determinations regarding significant household expenditure** |  |
|  | Respondent Alone | 562 (14·83) |
|  | Respondent And Husband/Partner | 2261 (59·66) |
|  | Husband/Partner Alone | 854 (22·53) |
|  | Missing | 113 (2·98) |
| 8 | **Whether mothers obtained permission for medical assistance** |  |
|  | Big Problem | 230 (6·07) |
|  | Not A Big Problem | 3559 (93·91) |
|  | Missing | 1 (0·03) |
| 9 | **Whether mother secured the necessary funds for treatment** |  |
|  | Big Problem | 626 (16·52) |
|  | Not A Big Problem | 3163 (83·46) |
|  | Missing | 1 (0·03) |
| 10 | **Whether mother expressed reluctance to seek medical help alone** |  |
|  | Big Problem | 800 (21·11) |
|  | Not A Big Problem | 2989 (78·87) |
|  | Missing | 1 (0·03) |
|  |  |  |
| **Organizational factors** | | |
|  | **Mother’s perception of distance to health facility** |  |
|  | Big problem | 426 (11·24) |
|  | Not a big problem | 3362 (88·71) |
|  | Missing | 2 (0·05) |
|  |  |  |
| **Community-level factors** | | |
| 1 | **Province** |  |
|  | Sumatera, | 975 (25.7) |
|  | Java and Bali | 1174 (31) |
|  | Nusa Tenggara | 339 (8.9) |
|  | Kalimantan | 336 (8.9) |
|  | Sulawesi | 571 (15.1) |
|  | Maluku and Papua | 395 (10.4) |
|  | Missing | 0 (0) |
| 2 | **Residency** |  |
|  | Urban | 1871 (49·37) |
|  | Rural | 1919 (50·63) |
|  | Missing | 0 (0) |
|  |  |  |
| **Policy-level factors** | | |
|  | **Covered by health insurance** |  |
|  | No | 1391 (36·70) |
|  | Yes | 2399 (63·30) |
|  | Missing | 0 (0) |

**Table S3· Results of univariate analyses of incomplete vaccination status based on vaccination card (N = 3,790)**

| **No** | **Characteristics** | **Fully Vaccinated**  **(n = 1,376; 36·3%)** | **Incomplete Vaccinated**  **(n = 2,414; 63·7%)** | **Univariate** | |
| --- | --- | --- | --- | --- | --- |
|  |  |  |  | **Odds ratio**  **(95% CI)** | **p-value** |
| 1 | **Mother's age (in years)** |  |  |  | 0.100* |
|  | 14–24 | 365 (26.5) | 649 (26.9) | 1.07 (0.92–1.27) |  |
|  | 24–34 | 748 (54.4) | 1239 (51.3) | Ref |  |
|  | 35–48 | 263 (19.1) | 526 (21.8) | 1.21 (1.01–1.44) |  |
|  | Missing | 0 (0) | 0 (0) |  |  |
| 2 | **Mother’s highest educational level** |  |  |  | 0·0001* |
|  | No or Primary Education | 279 (20·28) | 630 (26·1) | 1·39 (1·18–1·63) |  |
|  | Secondary and Higher Education | 1097 (79·72) | 1784 (73·9) | Ref |  |
|  | Missing | 0 (0) | 0 (0) |  |  |
| 3 | **Mother’s literacy** |  |  |  | 0·0000* |
|  | Cannot read at all | 39 (2·83) | 155 (6·42) | 2·35 (1·64–3·37) |  |
|  | Able to read | 1334 (96·95) | 2252 (93·29) | Ref |  |
|  | Missing | 3 (0·22) | 7 (0·29) |  |  |
| 4 | **Mother owns a mobile telephone** |  |  |  | 0·0000* |
|  | No | 235 (17·08) | 625 (25·89) | 1·69 (1·43–2·00) |  |
|  | Yes | 1138 (82·7) | 1788 (74·07) | Ref |  |
|  | Missing | 3 (0·22) | 1 (0·04) |  |  |
| 5 | **Mother’s occupation** |  |  |  | 0·0000* |
|  | Not Working | 760 (55·23) | 1241 (51·41) | 1·00 (0·86–1·18) |  |
|  | Clerk, Service, and Sales Person | 362 (26·31) | 589 (24·4) | Ref |  |
|  | Agriculture And Industrial Worker | 149 (10·83) | 356 (14·75) | 1·47 (1·16–1·85) |  |
|  | Professional And Manager | 105 (7·63) | 228 (9·44) | 1·33 (1·02–1·74) |  |
|  | Missing | 0 (0) | 0 (0) |  |  |
| 6 | **Mother’s media exposure** |  |  |  | 0·4413 |
|  | Less Than Once a Week | 657 (47·75) | 1184 (49·05) | 1·05 (0·92–1·20) |  |
|  | At Least Once A Week | 719 (52·25) | 1230 (50·95) | Ref |  |
|  | Missing | 0 (0) | 0 (0) |  |  |
| 7 | **Mother’s perception of distance to health facility** |  |  |  | 0·0007* |
|  | Big Problem | 123 (8·94) | 303 (12·55) | 1·46 (1·17–1·83) |  |
|  | Not A Big Problem | 1253 (91·06) | 2109 (87·37) | Ref |  |
|  | Missing | 0 (0) | 2 (0·08) |  |  |
| 8 | **Order of child birth** |  |  |  | 0·0000* |
|  | 1^st^, 2^nd^, and 3^rd^ | 1224 (88·95) | 1958 (81·11) | Ref |  |
|  | 4^th^ | 102 (7·41) | 249 (10·31) | 1·53 (1·20–1·94) |  |
|  | ≥5^th^ | 50 (3·63) | 207 (8·57) | 2·59 (1·88–3·56) |  |
|  | Missing | 0 (0) | 0 (0) |  |  |
| 9 | **Delivery location** |  |  |  | 0·0000* |
|  | Home delivery | 189 (13·74) | 708 (29·33) | 2·61 (2·18–3·12) |  |
|  | Facility delivery | 1187 (86·26) | 1706 (70·67) | Ref |  |
|  | Missing | 0 (0) | 0 (0) |  |  |
| 10 | **Number of antenatal care (in sessions)** |  |  |  | 0·0000* |
|  | 0−3 | 60 (4·36) | 348 (14·42) | 3·87 (2·90−5·16) |  |
|  | ≥4 | 1282 (93·17) | 1921 (79·58) | Ref |  |
|  | Missing | 34 (2·47) | 145 (6·01) |  |  |
| 11 | **Postnatal checks within 2 months** |  |  |  | 0·0002* |
|  | No | 382 (27·76) | 774 (32·06) | 1·32 (1·14–1·53) |  |
|  | Yes | 955 (69·4) | 1468 (60·81) | Ref |  |
|  | Missing | 39 (2·83) | 172 (7·13) |  |  |
| 12 | **Previous tetanus vaccination during pregnancy** |  |  |  | 0·0000* |
|  | No | 306 (22·24) | 747 (30·94) | 1·66 (1·42–1·94) |  |
|  | Yes | 1016 (73·84) | 1493 (61·85) | Ref |  |
|  | Missing | 54 (3·92) | 174 (7·21) |  |  |
| 13 | **Partner’s education level** |  |  |  | 0·0000* |
|  | No Education or Primary/Secondary Education | 339 (24·64) | 648 (26·84) | 1·14 (0·98–1·32) |  |
|  | Secondary and Higher Education | 1008 (73·26) | 1694 (70·17) | Ref |  |
|  | Missing | 29 (2·11) | 72 (2·98) |  |  |
| 14 | **Partner’s occupation** |  |  |  | 0·8238 |
|  | Clerk, Agriculture, Not working | 1243 (90·33) | 2186 (90·56) | 1·02 (0·82–1·28) |  |
|  | Professional and manager | 133 (9·67) | 228 (9·44) | Ref |  |
|  | Missing | 0 (0) | 0 (0) |  |  |
| 15 | **Total children under 5 years old** |  |  |  | 0·0000* |
|  | 1 And 2 | 974 (70·78) | 1485 (61·52) | Ref |  |
|  | ≥3 | 363 (26·38) | 756 (31·32) | 1·36 (1·18–1·59) |  |
|  | Missing | 39 (2·83) | 173 (7·17) |  |  |
| 16 | **Household wealth status** |  |  |  | 0·0001* |
|  | Lower and Middle | 1079 (78·42) | 2016 (83·51) | 1·39 (1·18–1·65) |  |
|  | Rich | 297 (21·58) | 398 (16·49) | Ref |  |
|  | Missing | 0 (0) | 0 (0) |  |  |
| 17 | **Covered by health insurance** |  |  |  | 0·4000 |
|  | No | 493 (35·83) | 898 (37·2) | 1·06 (0·92–1·22) |  |
|  | Yes | 883 (64·17) | 1516 (62·8) | Ref |  |
|  | Missing | 0 (0) | 0 (0) |  |  |
| 18 | **Residency** |  |  |  | 0·0000* |
|  | Urban | 749 (54·43) | 1122 (46·48) | Ref |  |
|  | Rural | 627 (45·57) | 1292 (53·52) | 1·37 (1·20–1·57) |  |
|  | Missing | 0 (0) | 0 (0) |  |  |
| 19 | **Province** |  |  |  | 0·0000* |
|  | Sumatera, | 259 (18.8) | 716 (29.7) | 2.89 (2.15–3.12) |  |
|  | Java, Bali | 568 (41.3) | 606 (25.1) | Ref |  |
|  | Nusa Tenggara | 135 (9.8) | 204 (8.5) | 1.42 (1.11–1.81) |  |
|  | Kalimantan | 151 (11) | 185 (7.7) | 1.15 (0.90–1.46) |  |
|  | Sulawesi | 185 (13.4) | 386 (16) | 1.95 (1.58–2.42) |  |
|  | Maluku and Papua | 78 (5.7) | 317 (13.1) | 3.81 (2.87-5.05) |  |
|  | Missing | 0 (0) | 0 (0) |  |  |
| 20 | **Individual responsible for making financial decisions within the household** |  |  |  | 0·0530* |
|  | Respondent Alone | 649 (47·17) | 1084 (44·9) | Ref |  |
|  | Respondent And Husband/Partner | 575 (41·79) | 978 (40·51) | 1·02 (0·88–1·17) |  |
|  | Husband/Partner Alone | 120 (8·72) | 267 (11·06) | 1·33 (1·05–1·69) |  |
|  | Missing | 32 (2·33) | 85 (3·52) |  |  |
| 21 | **Individual responsible for decisions about the mother's healthcare** |  |  |  | 0·8895 |
|  | Respondent Alone | 604 (43·9) | 1032 (42·75) | Ref |  |
|  | Respondent And Husband/Partner | 602 (43·75) | 1050 (43·5) | 1·02 (0·88–1·18) |  |
|  | Husband/Partner Alone | 142 (10·32) | 256 (10·6) | 1·05 (0·84–1·32) |  |
|  | Missing | 28 (2·03) | 76 (3·15) |  |  |
| 22 | **Determinations regarding significant household expenditure** |  |  |  | 0·4272 |
|  | Respondent Alone | 202 (14·68) | 360 (14·91) | Ref |  |
|  | Respondent And Husband/Partner | 813 (59·08) | 1448 (59·98) | 0·99 (0·82–1·21) |  |
|  | Husband/Partner Alone | 328 (23·84) | 526 (21·79) | 0·89 (0·72–1·12) |  |
|  | Missing | 33 (2·4) | 80 (3·31) |  |  |
| 23 | **Whether mothers obtained permission for medical assistance** |  |  |  | 0·8291 |
|  | Big Problem | 82 (5·96) | 148 (6·13) | Ref |  |
|  | Not A Big Problem | 1294 (94·04) | 2265 (93·83) | 0·96 (0·73–1·28) |  |
|  | Missing | 0 (0) | 1 (0·04) |  |  |
| 24 | **Whether mother secured the necessary funds for treatment** |  |  |  | 0·1149* |
|  | Big Problem | 210 (15·26) | 416 (17·23) | 1·16 (0·96–1·39) |  |
|  | Not A Big Problem | 1166 (84·74) | 1997 (82·73) | Ref |  |
|  | Missing | 0 (0) | 1 (0·04) |  |  |
| 25 | **Whether mother expressed reluctance to seek medical help alone** |  |  |  | 0·7080 |
|  | Big Problem | 286 (20·78) | 514 (21·29) | Ref |  |
|  | Not A Big Problem | 1090 (79·22) | 1899 (78·67) | 0·96 (0·82–1·14) |  |
|  | Missing | 0 (0) | 1 (0·04) |  |  |

Note:

*: Statistically significant (p value < 0·15)
